# P-594. Impact of Doxycycline Post-Exposure Prophylaxis on STI Incidence in a Community-Based Sexual Health Clinic in Miami-Dade County

**DOI:** 10.1093/ofid/ofaf695.808

**Published:** 2026-01-11

**Authors:** Ariana Johnson, Susanne Doblecki-Lewis

**Affiliations:** University of Miami- Department of Public Health Sciences, Miami, Florida; University of Miami Miller School of Medicine, Miami, Florida

## Abstract

**Background:**

The University of Miami Rapid Access Wellness Clinic, established in 2018, delivers same-day HIV prevention and sexual health services through one fixed site and mobile unit at five Miami-Dade locations. In April 2023, the clinic began offering doxycycline post-exposure prophylaxis (DoxyPEP) to reduce bacterial sexually transmitted infections (STIs). We evaluated changes in STI incidence before and after DoxyPEP initiation.

**Methods:**

We conducted a retrospective cohort study using clinical data from September 2018 to April 2025. For each client prescribed DoxyPEP, the post-DoxyPEP period began on the day of the first DoxyPEP prescription. The pre-DoxyPEP period extended from first recorded visit to the day before initiation. Incident cases of chlamydia and gonorrhea were identified via positive nucleic acid amplification test from urine, oral, or rectal samples. Early syphilis was diagnosed using positive/increased rapid plasma reagin (RPR) titer and clinical evaluation/adjudication of stage. Person-time was calculated for each period, and incidence rates were expressed per 100 person-years. Poisson regression models were fit separately for each STI, with event count as the outcome, log-person-time as the offset, and a binary pre/post indicator as the predictor. Incidence rate ratios (IRRs) and 95% confidence intervals (CIs) were derived.

**Results:**

Among 750 DoxyPEP recipients included, 87.9% identified as men who have sex with men, 63.6% as White, 76.0% as Hispanic or Latino, and 88.8% as male. The mean number of sexual partners reported in the past year was 8.7 (SD = 15.0). During the observation period, there were 304 chlamydia, 528 gonorrhea, and 189 early syphilis instances. Chlamydia incidence was 48% lower post-DoxyPEP (IRR = 1.48; 95% CI: 1.18–1.88; p < 0.001). Gonorrhea incidence increased by 33% post-DoxyPEP (IRR = 0.75; 95% CI: 0.63–0.88; p < 0.001). Early syphilis incidence did not significantly differ (IRR = 1.09; 95% CI: 0.82–1.47; p = 0.54).

**Conclusion:**

DoxyPEP appeared to reduce chlamydia incidence among individuals at risk in our cohort; the observed increase in gonorrhea underscores the need for continued surveillance. This real-world evaluation highlights the need for additional data regarding the impact of DoxyPEP in community-based sexual health programs.

**Disclosures:**

Susanne Doblecki-Lewis, MD, MSPH, Aspire Scientific: Medical writing support|Gilead Sciences, Inc.: Grant/Research Support|Gilead Sciences, Inc.: Medical writing support|Merck, Inc.: Grant/Research Support

